# Evaluation of the Association Between Congenital Cytomegalovirus Infection and Pediatric Acute Lymphoblastic Leukemia

**DOI:** 10.1001/jamanetworkopen.2022.50219

**Published:** 2023-01-09

**Authors:** Jennifer M. Geris, Mark R. Schleiss, Anthony J. Hooten, Erica Langer, Nelmary Hernandez-Alvarado, Michelle A. Roesler, Jeannette Sample, Lindsay A. Williams, David S. Dickens, Rajen J. Mody, Yaddanapudi Ravindranath, Kate L. Gowans, Matthew G. Pridgeon, Logan G. Spector, Heather H. Nelson

**Affiliations:** 1 Division of Epidemiology and Clinical Research, Department of Pediatrics, University of Minnesota, Minneapolis; 2 Institute for Molecular Virology, University of Minnesota, Minneapolis; 3 Division of Pediatric Infectious Diseases, Department of Pediatrics, University of Minnesota, Minneapolis; 4 Masonic Cancer Center, University of Minnesota, Minneapolis; 5 Division of Hematology/Oncology/Bone Marrow Transplantation, Department of Pediatrics, University of Iowa, Iowa City; 6 Division of Hematology-Oncology, Department of Pediatrics, Michigan Medicine, Ann Arbor; 7 Division of Hematology/Oncology, Department of Pediatrics, Wayne State University School of Medicine, and Children’s Hospital of Michigan, Detroit; 8 Department of Pediatric Hematology/Oncology, Beaumont Health, Royal Oak, Michigan; 9 Center for Cancer and Cell Biology, Van Andel Research Institute, Grand Rapids, Michigan; 10 Helen DeVos Children’s Hospital, Spectrum Health System, Grand Rapids, Michigan; 11 Division of Epidemiology and Community Health, University of Minnesota School of Public Health, Minneapolis

## Abstract

**Importance:**

Acute lymphoblastic leukemia (ALL) is the most common form of pediatric cancer, and a leading cause of death in children. Understanding the causes of pediatric ALL is necessary to enable early detection and prevention; congenital cytomegalovirus (cCMV) has recently been identified as a potential moderate-to-strong factor associated with risk for ALL.

**Objective:**

To compare the prevalence of cCMV infection between ALL cases and matched controls.

**Design, Setting, and Participants:**

In this population-based case-control study of ALL cases and matched controls, cases consisted of children aged 0 to 14 years between 1987 and 2014 with an ALL diagnosis identified through the Michigan Cancer Surveillance Program and born in Michigan on or after October 1, 1987. Cancer-free controls were identified by the Michigan BioTrust for Health and matched on age, sex, and mother’s race and ethnicity. Data were analyzed from November to May 2022.

**Exposures:**

cCMV infection measured by quantitative polymerase chain reaction in newborn dried blood spots.

**Main Outcomes and Measures:**

ALL diagnosed in children aged 0 to 14 years.

**Results:**

A total of 1189 ALL cases and 4756 matched controls were included in the study. Bloodspots were collected from participants at birth, and 3425 (57.6%) participants were male. cCMV was detected in 6 ALL cases (0.5%) and 21 controls (0.4%). There was no difference in the odds of cCMV infection comparing ALL cases with controls (odds ratio, 1.30; 95% CI, 0.52-3.24). Immunophenotype was available for 536 cases (45.1%) and cytogenetic data for 127 (27%). When stratified by subtype characteristics, hyperdiploid ALL (74 cases) was associated with 6.26 times greater odds of cCMV infection compared with unmatched controls (95% CI, 1.44-27.19).

**Conclusions and Relevance:**

In this case-control study of cCMV and pediatric ALL, cCMV was associated with increased risk of hyperdiploid ALL. These findings encourage continued research.

## Key Points

**Question:**

Is congenital cytomegalovirus (cCMV) infection associated with the development of acute lymphoblastic leukemia (ALL) during childhood?

**Findings:**

In this case-control study using newborn dried blood spots for 1189 ALL cases and 4756 matched controls, there was no significant difference in the odds of cCMV infection comparing ALL cases with matched controls. However, the odds of cCMV infection among hyperdiploid ALL cases were significantly greater compared with unmatched controls.

**Meaning:**

These findings suggest mixed evidence for an association between congenital CMV infection and ALL, specifically hyperdiploid disease, and continued research is warranted.

## Teaser

This case-control study evaluates the association between congenital cytomegalovirus infection and pediatric acute lymphoblasic leukemia.

## Introduction

Acute lymphoblastic leukemia (ALL) is the most common form of pediatric cancer, accounting for nearly 20% of all malignant neoplasms diagnosed in persons under 20 years of age.^1,2^ Highly penetrant genetic predispositions, such as Down syndrome, cause ALL in less than 5% of cases.^3^ Environmental risk factors have also been suggested, such as exposure to ionizing radiation, but the evidence is not conclusive.^4,5^ Largely, the cause of most ALL cases remains unknown.

Recently, cytomegalovirus (CMV) has emerged as a potential risk factor of ALL. CMV is a member of the herpesvirus family (HHV 5) and is capable of transplacental infection during pregnancy. In a report by Francis et al,^6^ congenital CMV (cCMV) infection was assessed in a population-based sample of newborn dried blood spots (DBS) from 268 ALL cases and 270 controls in California. Overall, cCMV was detected in 9.7% of ALL cases but only 3.0% of controls for a highly significant odds ratio (OR) of 3.71 (95% CI, 1.71-8.95). In a second study by Wiemels et al,^7^ congenital and early life, clinically recognized, CMV infection and subsequent ALL were investigated in population-based registries of Sweden. The hazard ratio (HR) of hematologic malignant neoplasm among children with any medically documented cCMV or early life acquired CMV was 11.2 (95% CI, 5.8-21.5).

Together, the 2 prior studies suggest prenatal CMV infection substantially increases risk of childhood ALL. If true, congenital and early life acquired CMV infection could represent the first modifiable risk factor for childhood ALL. As universal newborn screening for cCMV is in development, it is important to establish through replication whether CMV infection at birth is a risk factor for ALL. Therefore, this study assessed the association between cCMV and risk of childhood ALL.

## Methods

### Selection of Cases and Controls and Data Collection

Congenital CMV infection was assessed in a population-based case-control study through the Michigan BioTrust for Health (MBH). MBH is a Michigan Department of Health and Human Services program that oversees the use of residual newborn DBS collected shortly after birth along with routine linkage of the DBS repository to the birth and cancer registries. Cases consisted of children 0 to 14 years of age between 1987 and 2014 with an ALL diagnosis (International Classification of Diseases for Oncology, third edition code morphology 9835, 9836, and 9820) identified through the Michigan Cancer Surveillance Program and born in Michigan on or after October 1, 1987. Cytogenetic and molecular data on cases was abstracted through collaboration with 7 major Michigan children’s hospitals through an instrument programmed in REDCap. Controls were selected by MBH and matched 4:1 on year of birth, sex, and mother’s reported race and ethnicity. Sex and mother’s race and ethnicity were matched for efficient control of confounding while year of birth was matched to have equivalent windows of exposure. Birth characteristic data and mother’s race and ethnicity were obtained from linkage to the birth registry and included previously identified risk factors.^8,9^ Due to data suppression rules by MBH, any potential identifiable health information, such as birth year or mother’s and father’s age were categorized. The study was approved by the institutional review boards of the University of Minnesota and the Michigan BioTrust for Health. Parents of children with available DBS provided informed consent to MBH for the use of DBS in research studies. Informed consent was not required for this study as no cases or controls were contacted for additional information. This study is reported following the Strengthening the Reporting of Observational Studies in Epidemiology (STROBE) reporting guideline for observational studies.

### DNA Extraction and CMV Assay

Detection of CMV DNA in DBS was performed as described elsewhere,^10^ with some modifications. One 6-mm punch, equivalent to 28.27^2^mm in area, from the newborn DBS card was provided by MBH. DNA was extracted using the GenTegra GenSolve DNA Complete kit (GenTegra LLC). Briefly, the 6 mm punch was mixed with 609 μL lysis solution (GenTegra) and 11 μL of proteinase K (20 mg/mL) and incubated at 56 °C for 1.5 hours with agitation at 1400 rpm. Samples were transferred to a spin basket and centrifuged with a Recovery Solution (GenTegra). This was followed by DNA purification following the manufacturer’s protocol (GenTegra). Samples were eluted in 50 μL of elution buffer and stored at −20 °C.

Quantitative multiplex PCR was performed as described elsewhere.^11^ Briefly, 7 μL of eluate was used in a reaction volume of 35 μL using the LightCycle 480 PCR system (Roche). Primers and probes for the CMV-immediate early gene were used with neuroblastoma ras viral oncogene homolog (NRAS), in the same reaction as a housekeeping gene to confirm recovery of amplifiable DNA from DBS. PCR was run in triplicate, and a sample was considered positive if at least 2 of the 3 replicates were positive. Four cases (0.3%) and 13 controls (0.3%) did not have sufficient yield of genomic DNA (gDNA), as measured by NRAS, from the DBS and were scored indeterminate for cCMV. Three control DBS (0.1%) had CMV DNA below the predetermined limit of detection threshold and were scored equivocal. Copies of CMV were expressed as copies/mL of blood and copies/μg of genomic DNA, as previously described.^11^ Quantity of gDNA was measured by NRAS and expressed as picograms per PCR reaction (pg/PCR reaction).

### Statistical Analysis

A Pearson χ^2^ test was used to assess categorical data differences between cases and controls. Continuous variables were examined for a linear association with ALL and were categorized if nonlinear. To assess an association between cCMV and ALL, conditional logistic regression was used to construct univariable and multivariable models. Multivariable models were adjusted for mother’s age at birth, maternal diabetes, birth weight, categorical gestational age, and presence of congenital anomaly. Multivariable models were also stratified by age at diagnosis and subtype. Analysis of cases with available subtype data was conducted by matched and unmatched analysis using exact methods. All comparisons made between cases and controls used 2-sample *t* tests for continuous variables or Pearson χ^2^ or Fisher exact tests for categorical variables, and the level of significance was *P* = .05. All statistical analyses were performed using Stata/IC Version 15.1 (StataCorp LP). Data were analyzed from November to May 2022.

## Results

MBH identified 1199 eligible ALL cases and 4796 matched controls. Ten cases (0.83%) did not have a DBS available; therefore, the matched set (40 controls) was excluded. The final study population included 1189 cases and 4756 controls (Table 1). The mean (SD) age at ALL diagnosis was 4.5 years (3.3). The study population had a greater proportion of male children (3425 [57.6%]), and participants were predominately born between 1993 and 2002 (3205 [53.9%]). The mean (SD) birth weight of ALL cases (3448.6 [577] g) was higher than that of controls (3385.5 [573] g) (*t* = −3.39, *df* = 5943; *P* < .001). The distribution of mother’s categorical age was higher among ALL cases, with 153 (12.9%) having mothers who were aged 35 years or older compared with 547 controls (11.5%) (χ^2^_3_ = 51.83; *P* < .001). Despite matching on mother’s race and ethnicity, a higher proportion of cases had mothers who identified as White (987 [83%]) than controls (3959 [83.2%]) (χ^2^_4_ = 12.43; *P* = .01). There were no significant differences between mother’s level of education (χ^2^_3_ = 2.71; *P* = .44). Father’s categorical age at birth differed somewhat between cases and controls, with a greater proportion of fathers aged 35 years or older among cases (297 [25%]) than controls (994 [20.9%]) (χ^2^_3_ = 10.72; *P* = .01).

**Table 1.** Demographic Characteristics of ALL Cases and Controls

Characteristics	No. (%)		*P* value^a^
	ALL cases (n = 1189)	Controls (n = 4756)	
Age at diagnosis, mean (SD)	4.5 (3.3)	NA	
Birth year^b^			
1988-1992	212 (17.8)	848 (17.8)	.99
1993-1997	326 (27.4)	1304 (27.4)	
1998-2002	315 (26.5)	1260 (26.5)	
2003-2007	237 (19.9)	957 (20.1)	
2007-2012	99 (8.3)	387 (8.1)	
Sex^b^			
Female	504 (42.4)	2016 (42.4)	.99
Male	685 (57.6)	2740 (57.6)	
Mother’s age at birth, y			
<25	346 (29.1)	1592 (33.5)	<.001
25-34	679 (57.1)	2617 (55.0)	
≥35	153 (12.9)	547 (11.5)	
Unknown	11 (0.9)	0	
Mother’s race and ethnicity^b^			
Black	103 (8.7)	420 (8.8)	.01
Hispanic	56 (4.7)	231 (4.9)	
White	987 (83.0)	3959 (83.2)	
Other	26 (2.2)	122 (2.6)	
Unknown	17 (1.4)	24 (0.5)	
Mother’s level of education			
High school	556 (46.8)	2332 (49.0)	.44
Some beyond high school	326 (27.4)	1205 (25.3)	
College	293 (24.6)	1168 (24.6)	
Unknown	14 (1.2)	51 (1.1)	
Father’s age at birth, y			
<25	158 (13.3)	732 (15.4)	.01
25-34	590 (49.6)	2426 (51.0)	
≥35	297 (25.0)	994 (20.9)	
Unknown	144 (12.1)	604 (12.7)	
Father’s race and ethnicity			
Black	56 (4.7)	264 (5.6)	.52
Hispanic	45 (3.8)	214 (4.5)	
White	899 (75.6)	3500 (73.6)	
Other	25 (2.1)	114 (2.4)	
Unknown	164 (13.8)	664 (14.0)	
Father’s level of education			
High school	477 (41.1)	1900 (40.0)	.92
Some beyond high school	247 (20.8)	954 (20.1)	
College	298 (25.1)	1206 (25.4)	
Unknown	167 (14.1)	696 (14.6)	
**Pregnancy and birth characteristics**			
Pregnancy			
Weight gain during pregnancy, mean (SD), lb	31.4 (13.9)	31.0 (13.4)	.39
Smoking before or during pregnancy			
Yes	198 (16.7)	804 (16.9)	.96
No	968 (81.4)	3864 (81.2)	
Missing	23 (1.9)	88 (1.9)	
Alcohol use			
Yes	17 (1.4)	60 (1.3)	.90
No	1147 (96.5)	4595 (96.6)	
Missing	25 (2.1)	101 (2.1)	
Prepregnancy or gestational diabetes			
Yes	49 (4.1)	153 (3.2)	.12
No	1140 (95.9)	4603 (96.8)	
Chronic hypertension			
Yes	12 (1.0)	34 (0.7)	.30
No	1177 (99.0)	4.722 (99.3)	
Gestational hypertension			
Yes	9 (0.8)	25 (0.5)	.34
No	1180 (99.2)	4731 (99.5)	
Uterine/vaginal bleeding			
Yes	7 (0.6)	61 (1.3)	.04
No	1182 (99.4)	4695 (98.7)	
Previous cesarean delivery			
Yes	143 (12.0)	538 (11.3)	.49
No	1046 (88.0)	4218 (88.7)	
Birth			
Birth weight, mean (SD), g	3448.6 (577)	3385.5 (573)	<.001
Gestational age, wk			
<37	90 (7.6)	368 (7.7)	.32
≥37	1099 (92.4)	4379 (92.1)	
Unknown	0	9 (0.2)	
Method of delivery			
Vaginal			.54
Spontaneous	812 (68.3)	3357 (70.6)	
Forceps	25 (2.1)	99 (2.1)	
Vacuum	43 (3.6)	149 (3.1)	
Cesarean	300 (25.2)	1125 (23.7)	
Unknown	9 (0.8)	26 (0.6)	
Plurality of birth			
Single	1165 (98.0)	4663 (98.0)	.26
Twin	22 (1.8)	88 (1.9)	
Triplet	1 (0.1)	5 (0.1)	
Quadruplet	1 (0.1)	0	
Birth injury			
Yes	3 (0.3)	18 (0.4)	.51
No	1186 (99.7)	4738 (99.6)	
Congenital anomalies			
Yes	40 (3.4)	81 (1.7)	<.001
No	1116 (93.9)	4611 (97.0)	
Missing	33 (2.8)	64 (1.3)	
Kessner index			
Not collected	71 (6.0)	275 (5.8)	.54
Adequate	887 (74.6)	3449 (72.5)	
Intermediate	153 (12.9)	672 (14.1)	
Inadequate	73 (6.1)	334 (7.0)	
Unknown	5 (0.4)	26 (0.6)	
Kotelchuck index			
Not collected	51 (4.3)	204 (4.3)	.11
Adequate plus	395 (33.2)	1379 (29.0)	
Adequate	495 (41.6)	2091 (44.0)	
Intermediate	112 (9.4)	488 (10.3)	
Inadequate	89 (7.5)	407 (8.6)	
Unknown	47 (3.9)	187 (3.9)	

Abbreviation: ALL, acute lymphoblastic leukemia.

^a^ *P* values calculated by Pearson χ^2^ statistic for categorical variables or by 2-sided *t* test for continuous variables.

^b^ Indicates matching factor.

The mean (SD) weight gain during pregnancy was similar across mothers of cases (31.4 [13.9] pounds) and controls (31.0 [13.4] pounds) (*t* = −0.85, *df* = 5503; *P* = .39) (Table 1). There were no significant differences between mothers in smoking or alcohol use during pregnancy as recorded on birth certificates (χ^2^_2_ = 0.08; *P* = .96 and χ^2^_2_ = 0.21; *P* = .90, respectively). There was a slightly higher prevalence of prepregnancy or gestational diabetes among mothers of cases (49 participants [4.1%]) than controls (153 participants [3.2%]), but this difference was not significant (χ^2^_1_ = 2.37; *P* = .12). Among controls, there was a slightly higher prevalence of uterine or vaginal bleeding during pregnancy (61 participants [1.3%]) than cases (7 participants [0.6%]) (χ^2^_1_ = 4.05; *P* = .04). Mode of delivery and plurality of birth was similar across cases and controls (χ^2^_4_ = 3.10; *P* = .54 and χ^2^_3_ = 4.04; 0.26, respectively), with most births being single (98%, cases and controls) and spontaneous vaginal delivery (812 participants [68.3%] and 3357 participants [70.6%], respectively). There was a significantly higher prevalence of congenital anomaly among ALL cases (40 participants [3.4%]) compared with controls (81 participants [1.7%]) (χ^2^_2_ = 25.76; *P* < .001). There were no differences across Kotelchuck indexes (χ^2^_5_ = 8.86; *P* = .11).

**Figure 1.**
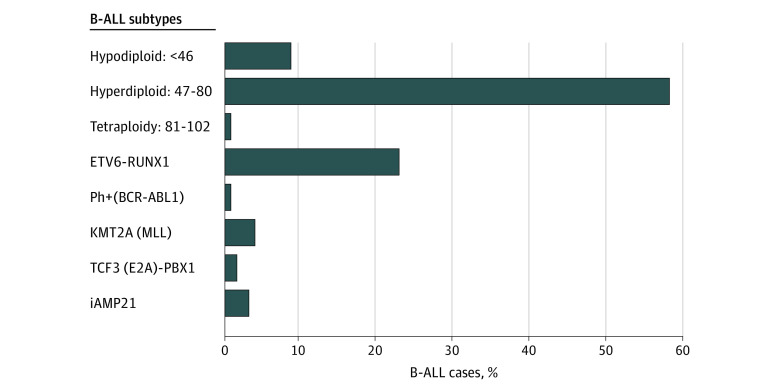
Distribution of B-Cell Acute Lymphoblastic Leukemia (B-ALL) Cytogenetic Subtypes Cytogenetic data were available for 127 (27%) of B-ALL cases.

Immunophenotype was available for 536 (45.1%) of cases; of those 62 (11.6%) were T-ALL and 474 (88.4%) were B-ALL (eTable in Supplement 1). Those with immunophenotype were more likely to be born after 1997 (446 participants [83.2%]) than those missing immunophenotype (205 participants [31.4%]) (χ^2^_4_ = 399.19; *P* < .001). Cases with subtype data were older at diagnosis (aged 5.4 years) compared with cases missing subtype (aged 3.8 years) (*P* < .001). However, cases with and without subtype were similar across sex (307 participants [57.3%] and 378 participants [57.9%] male, respectively) and mother’s race and ethnicity (435 participants [81.2%] and 552 participants [84.5%] White, respectively). Cytogenetic data were available for 127 (27%) of B-ALL cases. Among those with cytogenetic subtype, the distribution of B-ALL subtypes is shown in Figure 1. Hyperdiploid B-ALL was the most prevalent subtype (54 participants [58%]) followed by *ETV6-RUNX1* translocation (29 participants [23%]).

We detected cCMV DNA in 6 of 1189 of ALL cases (0.5%) and 21 of 4756 of controls (0.4%) (Table 2). The crude odds of cCMV infection were not statistically different between ALL cases and controls (OR, 1.14; 95% CI, 0.46-2.83). In the multivariable model, the odds of cCMV exposure did not differ between cases compared with controls (adjusted OR, 1.30; 95% CI, 0.52-3.24). Among B-ALL cases, the odds of cCMV appeared elevated compared with matched controls; however, this point estimate was also measured imprecisely (OR, 4.0; 95% CI, 0.56-28.40). There were no cCMV positive cases among recognized T-ALL. There were 2 hyperdiploid cases among those with subtype data that were cCMV positive. When we compared CMV prevalence among hyperdiploid ALL cases and their matched controls, the model did not converge due to lack of exposure among the matched controls. However, compared with all controls (4756 participants) in an unmatched analysis, hyperdiploid ALL cases (74 cases) were 6.26 times more likely to be CMV positive (95% CI, 1.44-27.19). We also assessed this outcome in an unmatched analysis including all matched controls of cases who had subtype data available (2144 participants) and found that the odds of cCMV exposure were 13.4 times greater among hyperdiploid cases compared with controls (95% CI, 1.25-83.21).

**Table 2.** Prevalence of cCMV in Dried Blood Spots of ALL Cases Compared With Matched Controls, by Diagnostic Characteristics

Characteristic	Proportion CMV positive, No./No. (%)		*P* value	OR (95% CI)^a^
	ALL cases	Controls		
Overall	6/1189 (0.5)	21/4756 (0.4)	.77	1.14 (0.46-2.83)^b^
Age at diagnosis, y				
0	0/51	1/204 (0.5)	>.99	NA
1-4	5/692 (0.7)	12/2768 (0.4)	.49	1.67 (0.59-4.73)
5-9	0/322	8/1288 (0.6)	.37	NA
10-14	1/124 (0.8)	0/494	.23	NA
B-ALL	2/474 (0.4)	2/1896 (0.1)	.17	4.0 (0.56-28.40)
T-ALL	0/62	3/248 (1.2)	>.99	NA
Ploidy (No. of chromosomes)				
Hypoploidy (<46)	0/11	0/44	NA	NA
Hyperploidy (47-80)	2/74 (2.7)	0/296	.04	NA (1.86-NA)^c^^,^^d^
Tetraploidy (81-102)	0/1	0/4	NA	NA

Abbreviations: ALL, acute lymphoblastic leukemia; cCMV, congenital cytomegalovirus; NA, not applicable; OR, odds ratio.

^a^ Odds ratio and 95% CI calculated by conditional logistic regression.

^b^ Multivariable model adjusted for mother’s age at birth, maternal diabetes, birth weight, categorical gestational age, and presence of congenital anomalies: OR, 1.30 (95% CI, 0.52-3.24); odds ratios and 95% CIs were calculated first by conditional logistic regression among hyperdiploid ALL cases and their matched controls, but the model did not converge.

^c^ An odds ratio calculated for hyperdiploid cases compared with all controls (n = 4756) using exact methods: OR, 6.26 (95% CI, 1.44-27.19).

^d^ Odds ratio calculated in an unmatched analysis for hyperdiploid cases compared with all controls who had cases with available subtype data (2144) using exact methods: OR, 13.37 (95% CI, 1.25 – 83.21).

**Figure 2.**
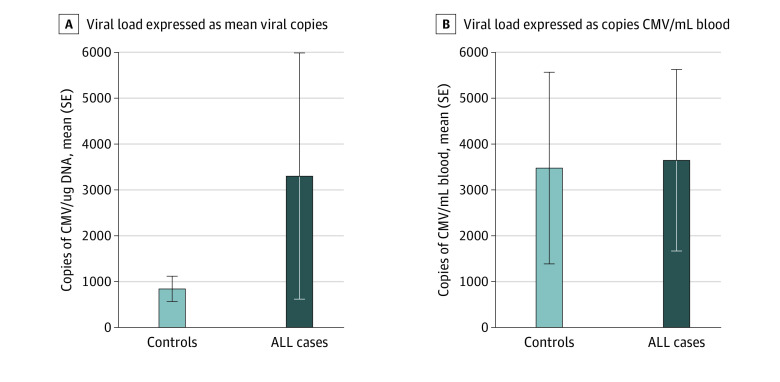
Mean Cytomegalovirus (CMV) Viral Load Among Acute Lymphoblastic Leukemia (ALL) Cases and Controls A, Viral load expressed as mean viral copies CMV/μg genomic DNA. *t* test *P* = .10. B, Viral load expressed as copies CMV/mL blood. *t* test *P* = .97.

Among those positive for cCMV DNA, the mean (SD) viral copies of CMV per mL of blood was not significantly different across cases (35 966.7 copies/mL [44 735.1]) and controls (34 368.1 copies/mL [88 339.6]) (*t* = −0.04, *df* =25; *P* = .97) (Figure 2). Cases had a higher mean (SD) viral load (3301.3 [6576.5] copies/μg) than controls (840.1 [1256.2] copies/μg), but this difference was not significant (*t* = −1.69, *df* = 25; *P* = .1). The mean (SD) quantity of gDNA used in each reaction was similar between cases (43 205 [46 409.6] pg/PCR reaction) and controls (56 719 [39 142.9] pg/PCR reaction) (*t* = 0.72, *df* = 25; *P* = .48).

In the stratified analysis of CMV infection, we did not detect any significant associations (Table 3). Among infants whose mothers had some education beyond high school, the prevalence of CMV was significantly different across cases and controls (Fisher exact *P* = .04); however, the crude OR was not significant (OR, 4.97; 95% CI, 0.84-34.07). We did not detect any differences in the odds of CMV infection by mother’s race and ethnicity, as all cases (6 participants) had White mothers.

**Table 3.** Congenital CMV Status Among ALL Cases and Controls, Stratified by Demographic Characteristics

Characteristic	No.				OR (95% CI)	*P* value^a^
	ALL cases		Controls			
	CMV positive (n = 6)	CMV negative (n = 1183)	CMV positive (n = 21)	CMV negative (n = 4732)		
Sex						
Female	3	501	9	2007	1.34 (0.23-5.38)	.72
Male	3	693	12	2725	0.98 (0.18-3.66)	.99
Birth weight (g)						
Low (<2500)	1	70	3	262	1.25 (0.02-15.80)	.99
Normal (2500-4000)	5	934	16	3927	1.31 (0.38-3.76)	.58
High (>4000)	0	179	2	543	NA	.99
Gestational age, wk						
<37	1	89	2	366	2.06 (0.03-39.83)	.48
≥37	5	1094	19	4357	1.05 (0.31-2.92)	.99
Mother’s age at birth, y						
<25	4	342	12	1578	1.54 (0.36-5.11)	.51
25-34	2	677	6	2610	1.29 (0.13-7.21)	.67
≥35	0	153	3	544	NA	.99
Mother’s race and ethnicity						
Black	0	103	5	415	NA	.59
Hispanic	0	56	1	230	NA	.99
White	6	981	13	3943	1.86 (0.58-5.24)	.24
Other	0	26	1	121	NA	.99
Mother’s level of education						
High school	2	554	17	2314	0.49 (0.05-2.08)	.56
Some beyond high school	4	322	3	1201	4.97 (0.84-34.07)	.04
College	0	293	1	1166	NA	.99
Father’s age at birth, y						
<25	1	157	3	729	1.55 (0.03-19.41)	.54
25-34	2	588	7	2416	1.17 (0.12-6.17)	.69
≥35	2	295	3	991	2.24 (0.19-19.63)	.33
Father’s race and ethnicity						
Black	0	56	1	263	NA	.99
Hispanic	0	45	1	213	NA	.99
White	4	895	9	3488	1.73 (0.39-6.22)	.32
Other	0	25	0	114	NA	NA
Father’s level of education						
High school	2	475	7	1892	1.14 (0.11-5.99)	.99
Some beyond high school	2	245	3	950	2.59 (0.21-22.67)	.27
College	0	298	1	1204	NA	.99
						
						
						

Abbreviations: ALL, acute lymphoblastic leukemia; CMV, cytomegalovirus; NA, not applicable; OR, odds ratio.

^a^ *P* values were calculated by Fisher exact test.

## Discussion

Congenital CMV infection has emerged as a potential modifiable risk factor of pediatric ALL. In response to the 2 extant studies of the topic by Francis et al^6^ and Wiemels et al,^7^ we conducted a large, population-based case-control study of cCMV infection and pediatric ALL. In the entire study of 1189 ALL cases and 4756 controls we did not detect an association between leukemia and exposure to cCMV infection in the main analysis. However, among hyperdiploid ALL cases, the odds of being cCMV positive were 6 times greater than unmatched controls. Later in this section, we discuss in brief the basis for investigating cCMV and ALL and compare our findings with those of Francis et al^6^ and Wiemels et al.^7^

There is a growing body of evidence of an infectious cause for pediatric ALL, and there are 3 main hypotheses on the nature of this pathogenesis: Greave’s delayed infection hypothesis, Kinlen’s population mixing hypothesis’, and Smith’s hypothesis of direct transformation by an infectious agent.^12,13,14,15^ Although none of these hypotheses anticipated an infection, such as CMV, which modulates the immune system for future infections, among existing hypotheses CMV best fits the criteria of Smith’s, which states the infectious agent causing ALL should possess: (1) ability to induce genomic instability; (2) specific effects on B lymphocytes; (3) higher rates of infection in lower socioeconomic status regions; (4) limited general oncogenic potential; (5) minimal symptoms associated with primary infection; and (6) ability to cross the placenta and infect the fetus, but not cause severe abnormalities.^15^

CMV is capable of causing direct chromosomal breakage in congenital infection, which is likely associated with its teratogenic properties,^16^ and encodes several proteins that modulate cell cycle control and the host DNA damage response.^17^ The association between cCMV and B lymphocytes are CD34^+^ cells, which are early hematopoietic progenitor cells in bone marrow,^18,19^ and the cell type in which CMV establishes latency. A study by Albano et al^20^ investigated the impact of cCMV on hematopoietic progenitor cell concentrations in cord blood and found among infants with cCMV infection, CD34^+^ cell populations were roughly 2.6 times greater than those of matched controls. This suggests a mechanism by which cCMV increases the risk of ALL by encouraging proliferation of cells vulnerable to transformation. Collectively, these observations support the plausibility of CMV being involved in the cause of ALL. Aside from this plausibility, 2 independent studies have suggested an association between cCMV infection and ALL.^6,7^

The initial study by Francis et al^6^ had a study design most similar to ours. The authors conducted a case-control study of newborn DBS from 268 ALL cases and 270 cancer-free controls. In comparison, our case-control study was nearly 10 times larger with a similarly strong, population-based study design using newborn DBS to capture prenatal CMV exposure. The rates of CMV DNA positivity were different in our 2 studies, with the positive prevalence 7.5 times higher in the controls from Francis et al^6^ compared with our controls. This raises the possibility of technical differences. One major difference was the use by Francis et al^6^ of droplet digital PCR to detect CMV DNA, while we used a quantitative PCR method optimized for detection of CMV DNA in newborn DBS.^11^ Although droplet digital PCR has been shown to have increased precision over quantitative PCR in certain applications, the methods have similar sensitivity.^21,22^ However, contaminants that originate from the Guthrie card could influence the sensitivity of the quantitative PCR assay. Another possibility is the variation in quantity of starting material. Francis et al^6^ reported using a quarter of a Guthrie spot, equivalent to about 33.2 mm^2^ area, which was more than the punch area of 28.27^2^ mm used in our study.^23^ Therefore, the likelihood of detecting cCMV could increase with the amount of material sampled. Although we cannot be certain what is driving the differences in CMV DNA prevalence at birth in the 2 studies, cCMV infection is detected in 0.45% of newborn DBS, which is consistent with our results.^11^ Furthermore, universal screening in high income countries have shown cCMV prevalence is consistently 0.6%.^24,25^

An outstanding question is whether the severity of cCMV is associated with future ALL risk. In Wiemels et al,^7^ both congenital and early life acquired CMV infection was evaluated for future risk of hematologic malignant neoplasm in population-based registries of Sweden. Through passive screening, the prevalence of clinically recognized CMV infection at birth was 0.0066% in noncases and 0.088% in children who later received a diagnosis of hematologic malignant neoplasm (HR, 14.8; 95% CI, 4.8-45.9). Congenital CMV infection is clinically recognized in 10%-15% of all infected infants, as the majority of babies are asymptomatic.^26,27^ Therefore, the CMV infections investigated in Wiemels et al were likely severe.^28^ We do not know who in our study had a clinically recognized cCMV infection; however, we observed higher CMV levels in cases vs controls. Further investigation into this association is necessary.

Our findings suggest a CMV-ALL association may be specific to hyperdiploid ALL, consistent with recent reports from diagnostic specimens.^29^ Cytogenetic subtype was available for 21% of cases. When stratified by ALL subtype, we found hyperdiploid ALL cases had 6.26 the odds of cCMV compared to all controls in our study, albeit based on only 2 exposed cases. Furthermore, hyperdiploid cases had 13.4 the odds of cCMV when compared with all controls who had cases with subtype data. High hyperdiploid ALL (generally defined as 50-67 chromosomes) was very recently shown to be associated with CMV by Gallant et al^29^ in case-only analyses. Half of all bone marrow biopsies were CMV DNA positive, and bone marrow biopsies from B-cell ALL cases were more likely to be CMV positive compared with T-cell ALL (OR 1.63; 95% CI, 0.88-3.06). Considering just B-cell ALL, the biopsies from high hyperdiploid ALL cases were 1.7 times more likely to be CMV positive than *ETV6-RUNX1* ALL, and 2.71 times more likely to be in the upper tertile of CMV-load (95% CI, 1.34-4.73).^29^ Our results, in combination with those from Gallant et al, strongly suggest CMV is associated specifically to hyperdiploid ALL.

### Strengths and Limitations

Our study had several strengths. First, this study was a very large, population-based case-control study of 1189 ALL cases and 4756 controls, all with DBS obtained immediately after birth, and nearly 10 times larger than the prior study. Second, data linkage through the MBH enabled us to examine potential associations with birth characteristics and parental demographics and leukemia. Lastly, the use of nested samples taken shortly after birth make the temporality between measurement of cCMV and development of ALL clear. Since samples were collected immediately following birth, any detectable DNA would have been passed in utero. Furthermore, this is an advantage over the study by Wiemels et al, since they used medical records, which reported only clinically recognized CMV disease.

A limitation of our study is this was a largely White population, which limited our ability to look in subgroups defined by demographics. Another limitation was Michigan DBS were stored at an ambient temperature, while DBS specimens collected after 2009 in the study by Francis et al^6^ were at −20 °C. This could explain why we had a lower prevalence of CMV DNA, but ambient temperature has not been previously associated with lower quality^30^ and our estimated prevalence was in line with other reports.^31^ Additionally, immunophenotype and cytogenetic subtype was available for a small portion of cases which contributed to imprecision of our point estimates.

## Conclusions

This case-control study did not find evidence of an association between cCMV infection and pediatric ALL in our primary analysis. However, we detected an association among hyperdiploid ALL cases using small numbers. The partial support given here to the association of cCMV with ALL, previously seen in 2 epidemiologic studies, and its accumulating biologic plausibility suggest the need for continued research. Minnesota and Ontario jurisdictions have recently initiated universal cCMV screening, which may present locales to examine this emerging association further.^32,33^ Future work may also consider incorporating DNA methylation to help establish a mechanistic link between cCMV and ALL. Finally, work that also considers early childhood infections is needed to understand the association between this prevalent virus and ALL.

## Notes

Supplement 1. **eTable. **Comparison of Cases With Available Leukemia Subtype to Cases Without, by Demographic Characteristics

Supplement 2. Data Sharing Statement
