# Supplementary material for: Evaluation of the Association Between Congenital Cytomegalovirus Infection and Pediatric Acute Lymphoblastic Leukemia
Source: JAMA Netw Open. 2023 Jan 9;6(1):e2250219. doi: 10.1001/jamanetworkopen.2022.50219 (PMC9856744; doi:10.1001/jamanetworkopen.2022.50219)
Supplement: Supplement 1. — eTable. Comparison of Cases With Available Leukemia Subtype to Cases Without, by Demographic Characteristics [file jamanetwopen-e2250219-s001.pdf]

Supplemental Online Content

Geris JM, Schleiss MR, Hooten AJ, et al. Evaluation of the Association Between Congenital Cytomegalovirus Infection and Pediatric Acute Lymphoblastic Leukemia. *JAMA Netw Open*. 2023;6(1):e2250219. doi:10.1001/jamanetworkopen.2022.50219

eTable. Comparison of Cases With Available Leukemia Subtype to Cases Without, by Demographic Characteristics

This supplemental material has been provided by the authors to give readers additional information about their work.

© 2023 Geris JM et al. *JAMA Network Open*

eTable. Comparison of Cases With Available Leukemia Subtype to Cases Without, by Demographic Characteristics

	Cases with subtype		Cases without subtype		
**Characteristics**	(n=536)	%	(n=653)	%	p-value
Mean age at diagnosis (SD)	5.4 (3.6)		3.8 (2.8)		<0.001
Birth year					<0.001
1988-1992	16	3.0%	196	30.0%	
1993-1997	74	13.8%	252	38.6%	
1998-2002	168	31.3%	147	22.5%	
2003-2007	182	34.0%	55	8.4%	
2007-2012	96	17.9%	3	0.5%	
Sex					
Female	229	42.7%	275	42.1%	
Male	307	57.3%	378	57.9%	
Mean birth weight, grams (SD)	3418.3 (571.0)		3473.4 (580.7)		
Gestational Age					0.063
<37 weeks	49	9.1%	41	6.3%	
37+ weeks	487	90.9%	612	93.7%	
Mother's age at birth, years					0.43
<25	148	27.6%	198	30.3%	
25-34	306	57.1%	373	57.1%	
35+	75	14.0%	78	11.9%	
Unknown	7	1.3%	4	0.6%	
Mother's race/ethnicity					0.23
White	435	81.2%	552	84.5%	
Black	52	9.7%	51	7.8%	
Other	9	1.7%	17	2.6%	
Hispanic	31	5.8%	25	3.8%	
Unknown	9	1.7%	8	1.2%	

A total of 536 cases had immunophenotype subtype available for analysis (B-ALL: n=474; T-ALL: n=62). P-values calculated by Pearson's Chi-Square statistic for categorical variables or by two-sided t-test for continuous variables; Abbreviations: SD – standard deviation.

© 2023 Geris JM et al. *JAMA Network Open*
