# Supplementary material for: Evaluation of the Association Between Congenital Cytomegalovirus Infection and Pediatric Acute Lymphoblastic Leukemia
Source: JAMA Netw Open. 2023 Jan 9;6(1):e2250219. doi: 10.1001/jamanetworkopen.2022.50219 (PMC9856744; doi:10.1001/jamanetworkopen.2022.50219)
Supplement: Supplement 2. — Data Sharing Statement [file jamanetwopen-e2250219-s002.pdf]

Data Sharing Statement

Geris. Evaluation of the Association Between Congenital Cytomegalovirus Infection and Pediatric Acute Lymphoblastic Leukemia. *JAMA Netw Open*. Published January 09, 2023. doi:10.1001/jamanetworkopen.2022.50219

Data
Data available: Yes
Data types: Other (please specify)
Additional Information: The data that support the findings of this study are available from Michigan BioTrust for Health (MBH) but restrictions apply to the availability of these data, which were used under data use agreements for the current study, and so are not publicly available. Data are however available from the authors upon reasonable request and with permission of MBH.
How to access data: https://www.michigan.gov/mdhhs/adult-child-serv/childrenfamilies/hereditary/biotrust/michigan-biotrust-for-health-dried-blood-spot-research
BioTrust@Michigan.gov
When available: With publication

Supporting Documents
Document types: None

Additional Information
Who can access the data: Researchers whose proposed use of the data has been approved
Types of analyses: For specified purpose
Mechanisms of data availability: After approval of a proposal and with signed data access agreement by MBH
